# Effects of Analgesics on Self-Reported Physical Function and Walking Ability in People With Hip or Knee Osteoarthritis: A Systematic Review and Meta-Analysis

**DOI:** 10.1093/ptj/pzad160

**Published:** 2023-11-19

**Authors:** Silje H Sveaas, Geir Smedslund, David A Walsh, Hanne Dagfinrud

**Affiliations:** Department of Nutrition and Public Health, Faculty of Health and Sport Sciences, University of Agder, Kristiansand , Norway; Norwegian National Advisory Unit on Rehabilitation in Rheumatology, Department of Rheumatology, Diakonhjemmet Hospital, Oslo, Norway; Pain Centre Versus Arthritis, Faculty of Medicine and Health Sciences, University of Nottingham, Nottingham, United Kingdom; Norwegian National Advisory Unit on Rehabilitation in Rheumatology, Department of Rheumatology, Diakonhjemmet Hospital, Oslo, Norway

**Keywords:** Analgesics, Hip Osteoarthritis, Knee Osteoarthritis, Physical Function, Walking Ability

## Abstract

**Objective:**

Hip and knee osteoarthritis are among the leading causes of global disability, and one of the main aims of the management is to improve physical function. The objective of this review was to investigate the effect of analgesics on physical function (self-reported physical function and walking ability).

**Methods:**

A systematic review and meta-analysis of the findings were performed. Randomized controlled trials investigating the effect of analgesics on self-reported physical function and walking ability were included. Analgesics were orally administered acetaminophen, nonsteroidal antiinflammatory drugs (NSAIDs), or opioids. Data were pooled in a random-effects model, and the standardized mean difference (SMD) with 95% CI was calculated (SMDs: 0.2–0.4 = small, 0.5–0.7 = medium, and ≥0.8 = large effect sizes). The quality of the evidence was evaluated according to the Grading of Recommendations Assessment, Development, and Evaluation approach.

**Results:**

A total of 1454 studies were identified, of which 33 were included. On self-reported physical function, the results showed low- to moderate-quality evidence for a small beneficial effect of acetaminophen (SMD = −0.13 [95% CI = −0.26 to 0.00]), NSAIDs (SMD = −0.32 [95% CI = −0.37 to −0.27]), or opioids (SMD = −0.20 [95% CI = −0.32 to −0.09]). There was moderate-quality evidence for a small effect of NSAIDs on pain during walking (SMD = −0.34 [95% CI = −0.45 to −0.23]).

**Conclusion:**

In people with hip or knee osteoarthritis, there was low- to moderate-quality evidence for small beneficial effects of analgesics on physical function and walking ability.

**Impact:**

Analgesics may improve physical function by reducing pain during exercise and walking.

## Introduction

Hip and knee osteoarthritis (OA) are among the leading causes of global disability.^1^ As no cure for OA is available, the management strategies aim to reduce symptoms and improve physical function.^2^^,^^3^ Physical function is defined as the ability to perform both basic and instrumental activities of daily living, and OA pain and stiffness are the main reasons given for reduced physical function in this group.^4^ Physical function has multiple dimensions, including how much a person can do, how easy it is, and how painful it is. In people with knee or hip OA, physical function is commonly measured by self-reported questionnaires (eg, Western Ontario and McMaster Universities Osteoarthritis Index).^5^ Western Ontario and McMaster Universities Osteoarthritis Index has a subscore for physical function with 17 items (descending stairs, ascending stairs, rising from sitting, standing, bending, walking, getting in/out of a car, shopping, putting on socks, taking off socks, rising from bed, lying in bed, getting in/out of a bath, sitting, getting on/off a toilet, heavy domestic duties, light domestic duties). Furthermore, physical function is commonly measured by measures of distance, speed, or pain during a standardized functional activity (eg, walking).^6^ In fact, a cross-sectional study of 500 people with OA showed that already at the age of 40 years, they had significantly poorer walking ability than their peers without arthritis.^7^

Core elements in the management of hip and knee OA are physical therapy and pharmacological treatment.^2^^,^^3^ Exercise is highlighted as the most important part of physical therapy as regular exercise may moderate the development of OA and improve physical function and quality of life for this group.^2^^,^^8^ Exercise is defined as a type of physical activity that is planned, structured, and repetitive with the purpose of improving or maintaining physical fitness.^9^

Hence, although the main aim of physical therapy for patients with hip or knee OA is to improve physical function and limit disability,^8^ the main aim of pharmacological treatment of OA is to relieve symptoms by analgesics. Oral nonsteroidal antiinflammatory drugs (NSAIDs) are strongly recommended for people without contraindications, while acetaminophen and opioids are conditionally recommended.^2^ Safe use of NSAIDs requires appropriate risk assessment and inclusion of gastroprotective strategies, and long-term use of opioids is associated with a high risk of toxicity and dependence.^2^ NSAIDs have analgesic effects by blocking cyclooxygenase enzyme activity and thereby reducing prostaglandin production,^10^ with actions that predominate locally within the joint. Acetaminophen has local analgesic effects by blocking cyclooxygenase enzyme activity^11^ but also act through mechanisms in the central nervous system,^12^ while opioids are predominantly centrally acting.^13^

A recent network meta-analysis concluded that exercise has similar effects on physical function and pain as do oral NSAIDs and acetaminophen in knee or hip OA.^14^ Exercise may have several positive additional effects, such as lowering the risks of all-cause mortality, cardiovascular diseases, type 2 diabetes, and cancer and improving bone health, cognition, sleep, and quality of life.^15^ Exercise has low risk of adverse advents, but it is time-consuming and injuries during activity might occur.

Pain and reduced physical function are well-known barriers to exercise in people with hip or knee OA,^16^ and use of analgesics prior to exercising is recommended as a disease-specific facilitator for exercise.^17^ Analgesics are readily available over the counter and frequently prescribed for this patient group.^2^^,^^3^ Previous systematic reviews on the effect of analgesics in people with hip or knee OA have investigated pain as the main outcome,^18–21^ but 2 systematic reviews have reported a small effect of NSAIDs on physical function.^20^^,^^21^ Leopoldino et al ^18^ reported high-quality evidence that acetaminophen provides small effect on self-reported physical function, and da Costa et al^19^ reported a small effect of opioids on self-reported physical function. However, there is a lack of studies that have included other physical function outcomes. Therefore, the aim of this review was to summarize the evidence for the therapeutic effect of 3 frequently used analgesics (acetaminophen, NSAIDs, and opioids) on self-reported physical function and walking ability, in people with hip or knee OA.

## Methods

The study was designed as a systematic literature review with meta-analysis. The protocol for this systematic review is registered in the PROSPERO register of systematic reviews (CRD42021271446). The review group consisted of methodologists and topic experts.

### Data Sources and Searches

The search strategy was prepared in collaboration with a health care librarian who performed the systematic literature searches.

The searches were performed in the databases MEDLINE, Embase, and the Cochrane Library from inception until September 2021. The search strategy for original studies is shown in Supplementary File 1. To confirm the search for original studies, a search for systematic reviews was also conducted, using the same search strategy, but limited to reviews. The reference lists of relevant systematic reviews were screened to ensure that all relevant original studies were included.

### Study Selection

Parallel-group randomized controlled trials (RCTs), crossover RCTs, and quasi-RCTs and a 1-group pretest-posttest study investigating the effect of oral analgesics on physical function (self-reported or walking ability) were considered eligible for inclusion. Investigated analgesics were limited to oral medications within 3 analgesic classes: acetaminophen, NSAIDs, and opioids. Participant groups were limited to people with hip or knee OA. Studies with mixed participant groups where data from those with hip or knee OA could not be isolated were excluded. RCTs without a placebo group that did not receive any analgesics were excluded, except for occasional use of acetaminophen for ≤3 consecutive days, which often is permitted for other reasons than OA pain. Furthermore, studies were limited to reports on humans and reports published in the English language.

### Screening Process

One review author (S.H.S.) performed the initial screening of titles and abstracts against the eligibility criteria using the online screening tool Rayyan.^22^ All articles selected in this process were obtained in full text. All full-text articles were assessed independently by 2 review authors (S.H.S. and G.S.). Disagreement among review authors was discussed until consensus was reached.

### Data Extraction

The data extraction process was 2-fold. First 1 review author (S.H.S.) extracted data from the included studies, and then another review author (G.S.) checked the extracted data against data in the full-text article. This process was used both when extracting results and when assessing methodological quality. If there was uncertainty regarding the extracted data, this was discussed in the review group and agreement was reached for each case. A unified dataset was entered into Review Manager (version 5.4.1)^23^ both for results and methodological quality.

Data on the effect of analgesics on relevant outcome measures were collected from the studies. Both posttreatment scores and change scores with SDs were collected in accordance with the original study. Data were collected from the latest reported follow-up points. For studies with multiple intervention groups of the same medication class, but with different dosage or different medicines within the same class (such as different types of NSAIDs), we combined the groups using weighted means based on sample sizes in the groups to ensure that 1 individual participant only was included in 1 group.^24^

### Quality Assessment

Methodological quality was assessed using the Cochrane Collaboration risk-of-bias tool^25^ based on published material. Risk-of-bias assessments were made at the study level for random sequence generation, allocation concealment, blinding of participants and personnel, incomplete outcome data, selective reporting, and any other bias.

When possible, we evaluated the quality of the evidence across trials according to the Grading of Recommendation Assessment, Development, and Evaluation approach at the outcome level. Factors that could reduce the quality of evidence were risk of biases, inconsistency of results, indirectness of evidence, imprecision, and publication bias. The quality of evidence was divided into 4 categories—high, moderate, low, and very low—according to how certain we were that the estimate was true (high quality indicated high confidence).^26^

### Data Synthesis and Analysis

Meta-analyses were conducted to summarize the results from original studies when the data allowed this. For continuous variables, the standardized mean difference (SMD) with 95% CI was calculated. SMDs between 0.2 and 0.4 were considered to be small effect sizes, those from 0.5 to 0.7 were considered to be medium effect sizes, and those of ≥0.8 were considered to be large effect sizes.^27^ Due to clinical heterogeneity between the trials, we decided to use a random-effects model for all outcomes. The Cochran *Q* was used to test for heterogeneity, and the I^2^ index was used to estimate the percentage of variability in results across studies that was due to real differences and not due to chance. A *P* value of ≤.05 was considered statistically significant. Results not included in the meta-analysis were summarized in the text.

### Funding Source

No funders contributed to the design, execution, or interpretation of the results.

## Results

### Study Selection

**Figure 1:**
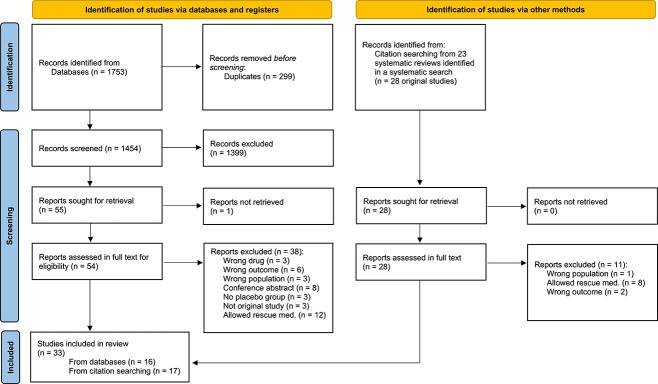
Flow diagram of the selection of trials. Med = medicine.

A total of 1454 records were identified by the searches. Of these, 82 records were assessed in full text for eligibility, and 33 of these were included in this systematic review (Fig. 1). Excluded trials with reason are shown in Supplementary File 2.

### Study Characteristics

The 33 included RCTs were published between 1999 and 2018 (Table). Nineteen studies included participants with knee OA, 13 studies included participants with hip or knee OA, and 1 study included only participants with hip OA. The total number of participants across the 33 studies was 19,092.

**Table:** Characteristics of Included Studies*^a^*

**Research Question and Pain Medication**	**Study**	**Design**	**Study Population** ^***b***^				**Pain Medication (Dose)**	**Duration**	**Risk of Bias** ^***c***^	**Main Results**
			**Group and Pain Descriptions**	**% Women**	**Mean Age (y)**	**Disease Duration (y)**				
Effect of analgesics on self-reported physical function										
Acetaminophen, NSAIDs, and opioids	DeLemos et al^41^	RCT	Opioids (*n* = 600), NSAIDs (*n* = 200), placebo (*n* = 200) Hip (26%), knee (74%) OA 81% ACR functional class II	60	60	8	Tramadol 100, 200, or 300 mg 1/d Celecoxib (200 mg 1/d)	12 wk	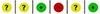	Significant positive effect of NSAIDs, no effect of tramadol on physical function
	Case et al^48^	RCT	NSAIDs (*n* = 25), acetaminophen (*n* = 29), placebo (*n* = 28) Knee OA Mean KL grade = 2 (SD = 1.0)	50	62		Diclofenac (75 mg 2/d) Acetaminophen (1 g 4/d)	12 wk	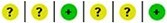	No effect of NSAIDs or acetaminophen on physical function
Acetaminophen	Prior et al^38^	RCT	Acetaminophen (*n* = 267), placebo (*n* = 275) Hip (18%), knee (82%) OA 72% ACR functional class II KL grade: 2 (60%), 3 (40%)	74	62		Acetaminophen (1300 mg 3/d)	12 wk	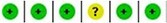	Significant positive effect of acetaminophen on physical function
	Herrero-Beaumont et al^53^	RCT	Acetaminophen (*n* = 108), placebo (*n* = 104) Knee OA KL grade: 2 (50%), 3 (40%), 2 or 3 (10%)	86	64	7	Acetaminophen (3 g/d)	6 mo	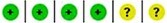	Significant positive effect of acetaminophen on physical function
	Altman et al^54^	RCT	Acetaminophen (*n* = 318), placebo (*n* = 165) Hip (20%), knee (80%) OA Moderate to severe OA pain (73%)	67	62		Acetaminophen (3900 or 1950 mg/d)	12 wk	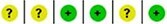	No effect of acetaminophen on physical function
	Miceli-Richard et al^50^	RCT	Acetaminophen (*n* = 405), placebo (*n* = 374) Knee OA Pain >30 (0–100) in past 24 h	75	70	2	Acetaminophen (4 g/d)	6 wk	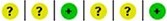	No effect of acetaminophen on physical function
NSAIDs	Lee et al^37^	RCT	NSAIDs (*n* = 258), placebo (*n* = 66) Hip (1%), knee (99%) OA Most participants: ACR functional class II	85	62		Polmacoxib (2 mg/d) or celecoxib (200 mg/d)	6 wk	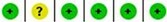	Significant positive effect of NSAIDs on physical function
	Essex et al^49^	RCT	NSAIDs (*n* = 254), placebo (*n* = 61) Knee OA Functional class: II (70%), III (25%)	64	60	6	Celecoxib (200 mg/d) or naproxen (500 mg 2/d)	6 wk	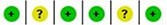	Significant positive effect of NSAIDs on physical function
	Conaghan et al^39^	RCT	NSAIDs (*n* = 233), placebo (*n* = 228) Knee OA Moderate pain	67	61		Celecoxib (100 mg 2/d)	12 wk	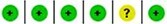	No effect of NSAIDs on physical function
	Essex et al^71^	RCT	NSAIDs (*n* = 249), placebo (*n* = 65) Knee OA Functional class: II (55%), III (45%)	80	58		Celecoxib (200 mg/d) or naproxen (500 mg 2/d)	6 wk	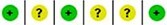	No effect of NSAIDs on physical function
	Puopolo et al^36^	RCT	NSAIDs (*n* = 428), placebo (*n* = 109) Hip (18%), knee (82%) OA ARA functional class: II (60%), III (12%)	75	63	7 (mean)	Etoricoxib (30 mg/d) or ibuprofen (2400 mg/d)	12 wk	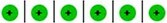	Significant positive effect of NSAIDs on physical function
	Wittenberg et al^72^	RCT	NSAIDs (*n* = 289), placebo (*n* = 75) Knee OA Pain ≥50 (0–100) at baseline	57	65	7 (mean)	Lumiracoxib (400 mg/d) or celecoxib (200 mg 2/d)	1 wk	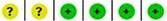	Significant positive effect of NSAIDs on physical function
	Sheldon et al^73^	RCT	NSAIDs (*n* = 1169), placebo (*n* = 382) Knee OA Pain ≥40 (0–100) at baseline	62	61	7 (mean)	Lumiracoxib (100 mg/d) or celecoxib (200 mg/d)	13 wk	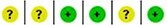	Significant positive effect of NSAIDs on physical function
	Lohmander et al^51^	RCT	NSAIDs (*n* = 747), placebo (*n* = 116) Hip (29%), knee (71%) OA Pain for at last 3 mo ACR functional class: I, II, or III	59	59		Naproxen (500 mg 2/d) or AZD3582 (750 mg 2/d)	6 wk	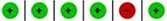	Significant positive effect of NSAIDs on physical function
	Gibofsky et al^74^	RCT	NSAIDs (*n* = 379), placebo (*n* = 96) Knee OA OA according to ACR criteria	67	63		Celecoxib (200 mg/d) or rofecoxib (25 mg/d)	6 wk	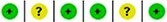	Significant positive effect of NSAIDs on physical function
	Makarowski et al^44^	RCT	NSAIDs (*n* = 349), placebo (*n* = 118) Hip OA Pain ≥40 (0–100) at baseline OA according to ACR definition	70	62	6 (mean)	Valdecoxib (5 or 10 mg/d) or naproxen (500 mg 2/d)	12 wk	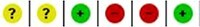	Significant positive effect of NSAIDs on physical function
	Gottesdiener et al^55^	RCT	NSAIDs (*n* = 557), placebo (*n* = 60) Knee OA ARA functional class: II (70%)	75	62	7 (mean)	Etoricoxib (5,10, 30, 60, or 90 mg/d)	6 wk	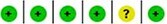	Significant positive effect of NSAIDs on physical function
	McKenna et al^75^	RCT	NSAIDs (*n* = 398), placebo (*n* = 200) Knee OA OA according to ACR criteria	66	62	8 (mean)	Celecoxib (100 mg 2/d) or diclofenac (50 mg 3/d)	6 wk	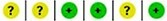	Significant positive effect of NSAIDs on physical function
	Ehrich et al^28^	RCT	NSAIDs (*n* = 498), placebo (*n* = 134) Hip, knee OA ARA functional class: II (70%)	71	62	10 (mean)	Rofecoxib (5, 12.5, 25, or 50 mg/d)	6 wk	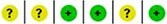	Significant positive effect of NSAIDs on physical function
	Yocum et al^46^	RCT	NSAIDs (*n* = 476), placebo *n* = 157) Hip (20%), knee (70%) OA OA confirmed by radiography	65	62	<5 in 50%	Meloxicam (3.75, 7.5, or 15 mg/d) or diclofenac (50 mg 2/d)	12 wk	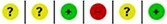	Significant positive effect of NSAIDs on physical function
	Bensen et al^76^	RCT	NSAIDs (*n* = 800), placebo (*n* = 203) Knee OA ACR functional class: I, II, or III	72	62	10 (mean)	Celecoxib (50, 100, or 200 mg 2/d) or naproxen (500 mg 2/d)	12 wk	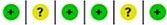	No effect of NSAIDs on physical function
Opioids	Kean et al^52^	RCT	Opioids (*n* = 392), placebo (*n* = 277) Knee OA, Moderate to severe pain OA according to ACR criteria	100	60		Tramadol (100, 200, or 300 mg/d)	12 wk	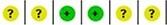	Significant positive effect of opioids on physical function
	Kivitz et al^43^	RCT	Opioids (*n* = 279), placebo (*n* = 91) Hip (20%), knee (80%) OA ACR functional class: II (76%–84%)	60	62		Oxymorphone ER (10, 40, or 50 mg/d)	2 wk	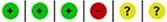	Significant positive effect of opioids on physical function
	Gana et al^47^	RCT	Opioids (*n* = 805), placebo (*n* = 205) Hip (25%), knee (75%) OA Pain ≥40 (0–100) OA according to ACR criteria	62	57		Tramadol ER (100, or 200, 300, or 400 mg/d)	12 wk	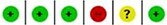	Significant positive effect of opioids on physical function
	Matsumoto et al^45^	RCT	Opioids (*n* = 367), placebo (*n* = 124) Hip (25%), knee (75%) OA Pain ≥40 (0–100) at baseline KL grade: ≥2	60	62	≥5 in 70%	Oxymorphone ER (40 or 80 mg/d or oxymorphone controlled release (40 mg/d)	3 wk	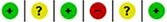	No effect of opioids on physical function
	Babul et al^40^	RCT	Opioids (*n* = 124), placebo (*n* = 122) Knee OA Pain ≥40 (0–100) at baseline OA according to ACR criteria	60	61	13 (mean)	Tramadol (100 mg/d and increased to 200 mg/d)	12 wk	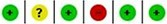	Significant positive effect of opioids on physical function
	Fleischmann et al^56^	RCT	Opioids (*n* = 63), placebo (*n* = 66) Knee OA Pain ≥2 (0–4, with 4 being worst) OA confirmed by radiography	60	62	8 (mean)	Tramadol (200–400 mg/d; 50-mg increments every 2 d to target dose)	12 wk	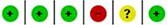	No effect of opioids on physical function
Effect of analgesics on walking ability	Couto et al^34^	RCT	NSAIDs (*n* = 409), placebo (*n* = 180) Hip (25%), knee (75%) OA KL grade: I–III Radiography-verified OA	70	61		Naproxen (660 or 440 mg/d)	1 wk	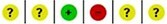	Significant effect of NSAIDs on walking speed
	Peeva et al^33^	Crossover study	19 patients with knee OA ARA class: II (90%)	64	60	9 (mean)	Naproxen (500 mg 2/d) Tramadol/acetaminophen (37.5 or 325 mg/d)	3 d	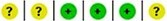	Significant effect of NSAIDs and tramadol/acetaminophen on pain during walking
	Moskowitz et al^30^	RCT	NSAIDs (*n* = 420), placebo (*n* = 110) Knee OA Pain ≥40 (0–100) at baseline OA according to ACR criteria ACR functional class: I–III	65	64	7.5 (mean)	Valdecoxib (10 mg) or rofecoxib (25 mg)	1 d	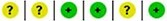	Significant positive effect of NSAIDs on pain during walking
	Weaver et al^31^	RCT	NSAIDs (*n* = 782), placebo (*n* = 196) Knee OA ACR functional class: II (64%)	70	62		Nabumetone (500 mg 2/d) or rofecoxib (12.5 mg/d)	6 wk	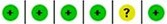	Significant positive effect of NSAIDs on pain during walking
	Wiesenhutter et al^32^	RCT	NSAIDs (*n* = 424), placebo (*n* = 104) Hip (20%), knee (70%) OA ARA functional class: II (57%)	70	62	8 (mean)	Etoricoxib (30 mg/d) or ibuprofen (2400 mg/d)	12 wk	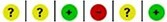	Significant positive effect of NSAIDs on pain during walking
	Golden et al^35^	RCT	NSAIDs (*n* = 162), acetaminophen (148), placebo (*n* = 155) Knee OA Radiography-confirmed OA	70	61		Naproxen (440 or 660 mg/d) Acetaminophen (4000 mg/d)	1 wk	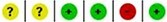	No effect of NSAIDs or acetaminophen on walking speed

^a^ ACR = American College of Rheumatology; ARA = American Rheumatism Association; /d = per day; ER = extended release; KL = Kellgren–Lawrence radiographic classification of osteoarthritis (OA) (1–4, with 4 being worst); NSAIDs = nonsteroidal antiinflammatory drugs.

^b^ Age, sex assigned at birth, and disease duration are given as brief descriptions of the experimental and control groups, and the numbers are not exact values.

^c^ Review authors’ judgment about each risk-of-bias item for each included study (1 = random sequence generation, 2 = allocation concealment, 3 = blinding of participants and personnel, 4 = incomplete outcome data, 5 = selective reporting, 6 = other bias).

Nineteen studies reported the effects of NSAIDs, 4 studies reported the effects of acetaminophen, 6 studies reported the effects of opioids, and 4 studies reported the effects of 2 different analgesics.

### Outcome Measures in the Included Trials

#### Self-Reported Physical Function

A total of 27 studies reported the effects of analgesics on physical function, and 26 of these measured physical function with the Western Ontario and McMaster Universities Osteoarthritis Index subscore for physical function.^5^ Higher scores on Western Ontario and McMaster Universities Osteoarthritis Index indicate worse functional limitations. One study^28^ measured physical function with the Short-Form Health Survey,^29^ in which a higher score indicates better function; therefore, the scores from this study were linearly transformed, so that a negative change also for this outcome measure indicated an improvement.

#### Walking Ability

Four studies^30–33^ reported the effects of analgesics on pain during walking, and 2^30^^,^^33^ of them measured pain during and after a walking test, while the 2 others used a self-reported question.^31^^,^^32^ Two studies^34^^,^^35^ reported the effect on walking speed, and both of them measured time used during a 50-foot walking test.

The duration of the intervention period varied between 1 day and 6 months, and the most common duration was 12 weeks. The included studies had a placebo group whose participants were not allowed to take any analgesics except acetaminophen for, at most, 3 consecutive days during the study period, for reasons other than OA pain.

### Risk of Bias in the Included Studies

The risks of bias in the included studies are shown in Supplementary File 3. One study was rated as low risk of bias for all items,^36^ and in 4 studies^31^^,^^37–39^ 1 of 6 items was rated as unclear risk. The majority of the studies had a low risk of selection bias as a proper random sequence generation was described. Likewise, most of the included studies did not provide sufficient details to judge if the allocation was concealed adequately and therefore had unclear risk of bias for this item. All the included studies were double blinded and had low risk of performance bias. Ten^32^^,^^34^^,^^40–47^ of 33 studies had high risk of attrition bias and 4^38^^,^^48–50^ had an unclear risk of attrition biases, mainly due to a high dropout rate and per protocol analyses. Few studies referred to a published protocol and were judged as having unclear risk of reporting bias, and 3 studies^35^^,^^44^^,^^51^ did not report the variability in the results and had high risk of reporting bias. Moreover, the included studies were considered as having low risk of other bias, but 5 studies had unclear risk of other biases due to reasons such as a combination of different datasets,^34^^,^^52^ scores for physical function not being collected before each treadmill walk,^33^ the use of a fixed dose regimen,^43^ and/or a selected study group.^53^

### The Effect of Analgesics on Self-Reported Physical Function

#### Acetaminophen

**Figure 2:**
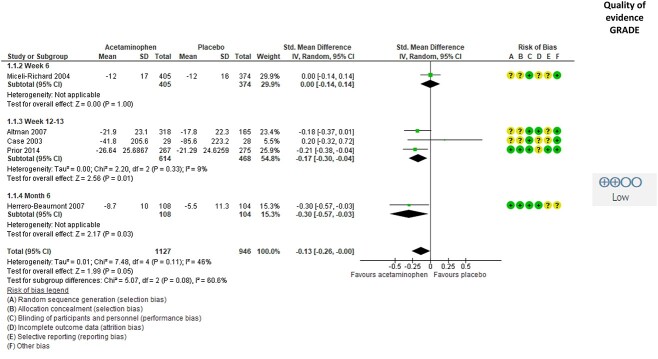
Effect of acetaminophen on self-reported physical function in people with hip or knee osteoarthritis. Values are shown as standardized mean difference with 95% CI. Downgrading to low-quality evidence was due to risk of selection bias and inconsistency across studies. GRADE = Grading of Recommendations Assessment, Development and Evaluation approach; IV = inverse variance.

Five studies^38^^,^^48^^,^^50^^,^^53^^,^^54^ evaluated the effect of acetaminophen on self-reported physical function, and all these provided data to the meta-analysis (Fig. 2). The results showed low-quality evidence for a small beneficial effect of acetaminophen on physical function (SMD = −0.13 [95% CI = −0.26 to −0.00]; *P* = .05) in patients with hip and knee OA. There was substantial unexplained heterogeneity (I^2^ = 46%) and high risk of selection bias, and the quality of the evidence was therefore downgraded to low.

#### Nonsteroidal Antiinflammatory Drugs

**Figure 3:**
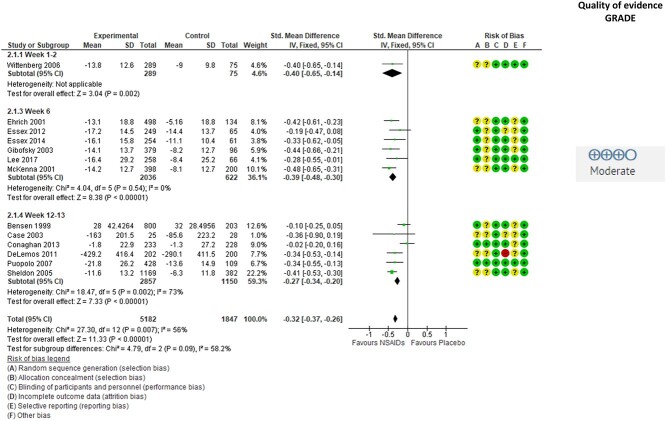
Effect of nonsteroidal antiinflammatory drugs (NSAIDs) on self-reported physical function in people with hip or knee osteoarthritis. Values are shown as standardized mean difference with 95% CI. Downgrading to moderate-quality evidence was due to risk of selection and reporting bias. GRADE = Grading of Recommendations Assessment, Development and Evaluation approach; IV = inverse variance.

A total of 17 studies evaluated the effect of NSAIDs on self-reported physical function, and 13 of these provided effect size data and were included in the meta-analysis (Fig. 3). The results showed moderate-quality evidence for a significant small beneficial effect of NSAIDs on physical function (SMD = −0.32 [95% CI = −0.37 to −0.26]; *P* = .007). For NSAIDs, the quality of the evidence was downgraded to moderate due to the risk of selection and reporting bias. There was low risk of publication bias as illustrated in the funnel plot in Supplementary File 3. However, there was substantial heterogeneity (I^2^ = 56%). In addition, the 4 studies^44^^,^^46^^,^^51^^,^^55^ that did not provide data to the meta-analysis reported a significant beneficial effect of NSAIDs on self-reported physical function.

#### Opioids

**Figure 4:**
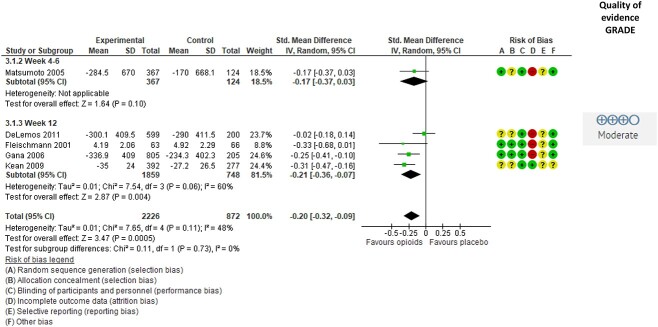
Effect of opioids on self-reported physical function in people with hip or knee osteoarthritis. Values are shown as standardized mean difference with 95% CI. Downgrading to moderate-quality evidence was due to attrition bias. GRADE = Grading of Recommendations Assessment, Development and Evaluation approach; IV = inverse variance.

Seven studies^40^^,^^41^^,^^43^^,^^45^^,^^47^^,^^52^^,^^56^ evaluated the effect of opioids on self-reported physical function, and 5 of these provided data to the meta-analysis (Fig. 4). The results showed moderate-quality evidence for a small beneficial effect of opioids on physical function in people with hip and knee OA (SMD = −0.20 [95% CI = −0.32 to −0.09]; *P* < .001). There was moderate heterogeneity (I^2^ = 48). The quality of evidence was downgraded to moderate due to a high risk of attrition bias, as there was a high dropout rate in the included trials. In addition, also the 2 studies^40^^,^^43^ not included in the meta-analysis showed a significant beneficial effect of opioids on self-reported physical function.

### The Effect of Analgesics on Walking Ability

**Figure 5:**
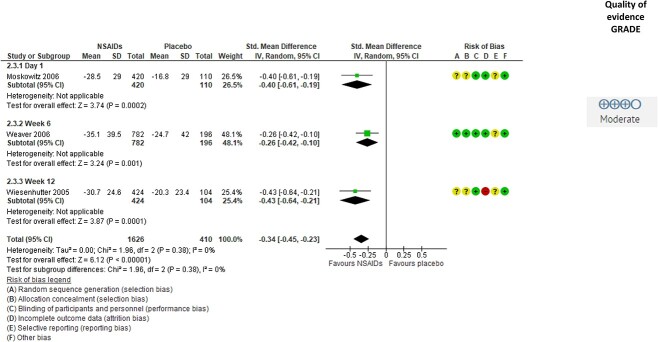
Effect of nonsteroidal antiinflammatory drugs (NSAIDs) on pain during walking in people with hip or knee osteoarthritis. Values are shown as standardized mean difference with 95%CI. Downgrading to moderate-quality evidence was due to attrition bias. GRADE = Grading of Recommendations Assessment, Development and Evaluation approach; IV = inverse variance.

Four studies^30–33^ evaluated the effect of analgesics on pain during walking and 3^30–32^ of these provided data suitable for meta-analysis, showing moderate-quality evidence for a small beneficial effect of NSAIDs (SMD = −0.34 [95% CI = −0.45 to −0.23]; *P* < .001) (Fig. 5). No heterogeneity was detected (I^2^ = 0). The quality of the evidence was downgraded from high to moderate due to attrition bias. One study that was not included in the meta-analysis^33^ supported a beneficial effect, finding a significant effect on pain during walking of both NSAIDs and a combination of opioids and acetaminophen. Two studies^34^^,^^35^ evaluated the effect of analgesics on walking speed, and 1 of these^34^ found a significant beneficial effect of NSAIDs compared to placebo, whereas the other^35^ found no significant effect of either NSAIDs or acetaminophen on walking speed.

## Discussion

The results of this systematic review showed low- to moderate-quality evidence for a small beneficial effect of NSAIDs, acetaminophen, and opioids on physical function in people with hip or knee OA. Beneficial effects were detected in people with hip or knee OA for self-report physical function outcomes, as well as specifically for effects on pain during walking. Nevertheless, the effect sizes were small, and side effects of analgesics should always be taken into consideration.^2^

To the best of our knowledge, this is the first systematic review reporting the impact of 3 different types of analgesic medication on physical function, including walking ability, in people with hip or knee OA. Our finding of small but significant effects of analgesics on physical function is in line with results reported in previous systematic reviews, indicating high-quality evidence for small to no clinically important effects of acetaminophen^18^ and small effects of NSAIDs^20^^,^^21^ and opioids^19^ on physical function in people with hip or knee OA. During the past decade, there has been a shift in the management of OA, from pain control to interventions aiming to improve physical function and general health.^17^ Exercise is recommended not only to reach the goal of improved physical function, but also due to numerous general health effects^17^ and positive effects on disease activity and symptoms.^57^ The result of this review supports the use of analgesics to increase physical function and thereby facilitate participation in exercise, which in turn may give a wide range of beneficial health effects.

The effect sizes for analgesics on self-reported physical function found in the current review (SMDs between 0.13 and 0.32) are smaller than those reported for the effect of an exercise program on physical function (SMD = 0.41) in people with hip OA in another systematic review.^58^ The meta-analysis on effects of exercise on physical function in hip OA showed that studies with interventions following American College of Sports Medicine exercise recommendations had larger effect sizes than studies not following these recommendations.^58^ Furthermore, a recent meta-analysis found that exercise had effects on physical function that were comparable to those of analgesic medications.^14^ Future studies should investigate whether the analgesic effects of exercise and medicine are additive or even synergistic. Meanwhile, current evidence supports a therapeutic emphasis on exercise, possibly supplemented and facilitated by analgesic use.

Use of analgesic medications might be limited by low efficacy to reduce pain, or by the risk of adverse events. For example, an increased risk of cardiovascular diseases following use of NSAIDs has been reported,^59^ and use of NSAIDs or opioids may be contraindicated in elderly patients with hip or knee OA.^2^ Nevertheless, analgesic medicine is recommended for people with hip or knee OA,^2^ and those who already use analgesics may be encouraged to take them 30 to 60 minutes before exercise.^60^ In addition, people with hip or knee OA are recommended to use analgesics only during a short time,^2^ and prescription of analgesics may provide a window of opportunity to commence exercise. Analgesics may reduce disease-related pain in people with hip or knee OA,^18–21^ and it has also been reported that they may reduce exercise-induced pain and delayed-onset muscle soreness in adults who are healthy.^61^ Especially in the initial phase of an exercise program, analgesic medication may be helpful to overcome exercise induced pain and thereby increase adherence to recommended exercise programs.

Our finding of a positive effect of analgesics on physical function is in line with a single 1-group pretest-posttest study that investigated whether optimal use of analgesics may enable people with knee OA with severe pain to exercise.^62^ In addition, this study reported that almost all the included people with knee OA reported negative attitudes toward use of analgesics as they worried about side effects and risk of addiction, but they became more positive after experiencing the positive effects of analgesics.^62^ This highlights that experiential learning during exposure to an intervention might change beliefs and attitudes, and the importance of concordant information provided about analgesic medications, and how they should be used to facilitate exercise.

Along with the positive effects of NSAIDs on physical function found, negative effects have been reported on muscle growth in young adults who are healthy.^61^^,^^63^ However, in patients with knee OA, a previous study have reported that NSAIDs used in conjunction with exercise might have a positive effect on muscle strength,^64^ and negative effects have not been demonstrated of NSAIDs used in conjunction with exercise on muscle mass,^64^ muscle protein synthesis,^65^ or cartilage turnover.^66^ In addition, positive effects of NSAIDs and acetaminophen on adaptations to strength exercises have also been shown in older adults.^67^ Hence, there might be beneficial effects of NSAIDs on adaptation to strength exercise in people with knee or hip OA, perhaps attributable to their pain-relieving effects. The negative effects of NSAIDs on adaptations to strength exercises in young adults who are healthy^61^^,^^63^ have been explained by the inhibition of cyclooxygenase activity, which is essential for muscle protein synthesis and thereby muscle growth.^61^ Effects of analgesics on muscle growth seem to be dependent of age, pain, and inflammatory status.

Our systematic review found a beneficial effect of opioids on physical function in people with hip or knee OA. Opioids might have analgesic efficacy of only limited duration in chronic pain,^68^ and any short-term benefits on physical function would need to be balanced against risks of adverse events or dependency. In summary, medications investigated in the current study have limited analgesic efficacy, and more potent analgesic agents may have greater potential to increase benefits of exercise on physical function in OA.

### Limitations

The literature search for this review was performed in only 3 databases, and although this is adequate according to the AMSTAR 2 appraisal tool for systematic reviews of health care interventions,^69^ more extensive search strategies might have identified additional studies. A search of reference lists of systematic reviews was also conducted, and as almost half of the included studies were identified during this process, the comprehensiveness of the search strategy may be questioned. However, since physical function was a secondary outcome measure in these studies it, was not listed as a keyword in the records, and that is probably the reason why they were not identified in the search of original studies. Overall, the screening of reference lists of systematic reviews strengthens the results of this review by increasing the likelihood that all relevant records were included.

The aim of the current systematic review was originally to also investigate the effect of analgesics on physical activity level and physiological responses to exercise (as stated in the protocol, [PROSPERO; CRD42021271446]), in addition to physical function, but there was a lack of research on these outcomes. Physical function may be considered as a prerequisite for the ability to physically active and further research is required to explore these topics.

Physical function was self-reported in most of the included studies, and measures of physical function might be confounded by pain severity, which is often incorporated into physical function questionnaire items.^70^ Hence, future studies should investigate the effect of analgesic on performance based physical function, including physical functions other than walking.

### Conclusion

The results of this systematic review suggest that analgesics may have a small beneficial effect on self-reported physical function and walking ability in people with hip or knee OA. Our findings lead us to suggest that analgesics may improve physical function by reducing pain during exercise and walking, and therefore have potential to increase physical activity in people with OA hip or knee OA. Future studies investigating the effects of analgesics on physical activity and exercise participation are warranted.

## Supplementary Material

2022-0566_R2_Supplementary_Files_tsr_pzad160

## Systematic Review Registration

The protocol for this systematic review is registered in the PROSPERO register of systematic reviews (CRD42021271446).
