# Supplementary material for: Effects of Analgesics on Self-Reported Physical Function and Walking Ability in People With Hip or Knee Osteoarthritis: A Systematic Review and Meta-Analysis
Source: Phys Ther. 2023 Nov 19;104(2):pzad160. doi: 10.1093/ptj/pzad160 (PMC10902557; doi:10.1093/ptj/pzad160)
Supplement: 2022-0566_R2_Supplementary_Files_tsr_pzad160 [file 2022-0566_r2_supplementary_files_tsr_pzad160.pdf]

Supplementary file 1

The search strategy used in Medline, Embase and the Cochrane Library

Search strategy original studies

----

1 osteoarthritis/ or hip osteoarthritis/ or knee osteoarthritis/

2 osteoarthr*.mp.

3 or/1-2

4 exercise/

5 exp kinesiotherapy/

6 exercise program*.mp.

7 exercise therap*.mp.

8 mobility exercise.mp.

9 muscle exercis*.mp.

10 physical exercis*.mp.

11 exp stretching exercise/

12 exp sport/

13 exp exercise/

14 exp fitness/

15 exp exercise test/

16 exp exercise tolerance/

17 exp pliability/

18 exp endurance/

19 exertion.mp.

20 exercise*.mp.

21 sport*.mp.

22 exp accelerometry/

23 accelerometer data.mp.

24 physical function*.mp.

25 exp energy metabolism/

26 exp recreation/

27 physical activit*.mp.

28 energy expendit*.mp.

29 training.mp.

30 exp physical education/

31 exp muscle strength/

32 exp muscle mass/

33 exp muscle growth/

34 muscle mass.mp.

35 muscle strength.mp.

36 muscular strength.mp.

37 muscle growth.mp.

38 muscular growth.mp.

39 or/4-38

40 exp nonsteroid antiinflammatory agent/

41 exp analgesic agent/

42 analgesic*.mp.

43 exp paracetamol/

44 acetaminophen.mp.

45 exp morphine/

46 exp oxycodone/

47 exp codeine/

48 exp tramadol/

49 or/40-48

50 and/3,39,49

51 limit 50 to (controlled clinical trial or randomized controlled trial)

52 exp randomized controlled trial/

53 ((randomized or randomised or controlled) adj2 (trial* or stud*)).mp.

54 RCT*.mp.

55 ((pre-experiment* or preexperiment*) adj2 (design* or trial* or stud* or analys*)).mp.

56 ((quasi-experiment* or quasiexperiment*) adj2 (design* or trial* or stud* or analys*)).mp.

57 ((quasi-randomised or quasi-randomized) adj2 (design* or trial* or stud* or analys*)).mp.

58 or/52-57

59 and/3,39,49,58

60 or/51,59

61 (animal experiment/ or exp animal/ or exp animal model/) not human/

62 (veterinary or animal or animals or rabbit or rabbits or rodent or rodents or rat or rats or mouse or mice or rabbit or rabbits or pig or pigs or porcine or pigeon* or horse* or equine or cow or cows or bovine or goat or goats or sheep or ovine or dog or dogs or canine or cat or cats or feline or dolphin*).ti.

63 60 not (61 or 62)

Search strategy reviews

1 osteoarthritis/ or hip osteoarthritis/ or knee osteoarthritis/

2 osteoarthr*.mp.

3 or/1-2

4 exercise/

5 exp kinesiotherapy/

6 exercise program*.mp.

7 exercise therap*.mp.

8 mobility exercise.mp.

9 muscle exercis*.mp.

10 physical exercis*.mp.

11 exp stretching exercise/

12 exp sport/

13 exp exercise/

14 exp fitness/

15 exp exercise test/

16 exp exercise tolerance/

17 exp pliability/

18 exp endurance/

19 exertion.mp.

20 exercise*.mp.

21 sport*.mp.

22 exp accelerometry/

23 accelerometer data.mp.

24 physical function*.mp.

25 exp energy metabolism/

26 exp recreation/

27 physical activit*.mp.

28 energy expendit*.mp.

29 training.mp.

30 exp physical education/

31 exp muscle strength/

32 exp muscle mass/

33 exp muscle growth/

34 muscle mass.mp.

35 muscle strength.mp.

36 muscular strength.mp.

37 muscle growth.mp.

38 muscular growth.mp.

39 or/4-38

40 exp nonsteroid antiinflammatory agent/

41 exp analgesic agent/

42 analgesic*.mp.

43 exp paracetamol/

44 acetaminophen.mp.

45 exp morphine/

46 exp oxycodone/

47 exp codeine/

48 exp tramadol/

49 or/40-48

50 and/3,39,49

51 limit 50 to systematic review

52 exp "systematic review"/

53 "systematic review".mp.

54 or/52-53

55 and/50,54

56 or/51,55

57 (animal experiment/ or exp animal/ or exp animal model/) not human/

58 (veterinary or animal or animals or rabbit or rabbits or rodent or rodents or rat or rats or mouse or mice or rabbit or rabbits or pig or pigs or porcine or pigeon* or horse* or equine or cow or cows or bovine or goat or goats or sheep or ovine or dog or dogs or canine or cat or cats or feline or dolphin*).ti.

59 56 not (57 or 58)

Supplementary file 2 List of excluded studies with reason

Study	Reason for exclusion
Search for original studies	
Report not retrieved in full text	
1. Fishman RL, Kistler CJ, Ellerbusch MT, et al. Efficacy and safety of 12 weeks of osteoarthritic pain therapy with once-daily tramadol (Tramadol Contramid OAD). *Journal of opioid management.* 2007;3(5):273-280.	Not available in full text
Excluded studies	
1. Altman R, Hochberg M, Kivitz A, Gibofsky A, Solorio D, Young C. Low-dose solumatrix meloxicam results in clinically meaningful improvements in pain in a phase 3 study of patients with osteoarthritis. *Osteoarthritis and Cartilage.* April 2016;24:S419-S420.	Conference abstract only
2. Kelly K, Greene A, Kuperwasser B, et al. Effects of tapentadol extended release on the western ontario and mcmaster universities osteoarthritis index (WOMAC) and pain intensity in patients with chronic osteoarthritis pain: Results of a randomized, phase 3, active- and placebo-controlled study. *Arthritis and Rheumatism*. 2009;10):850.	Conference abstract only
3. Birnbaum A, Runels T, Bean A, Hwang U. A comparison of analgesics on physical function in older veterans with osteoarthritis. *Journal of General Internal Medicine.* 2018;33(2 Supplement 1):88-89	Conference abstract only
4. Lapane KL, Yang S, McAlindon T, Eaton CB. Non-steroidal anti-inflammatory agents in reducing symptoms in knee osteoarthritis: Effectiveness over time. *Osteoarthritis and Cartilage.* 2014 2014;22:S208-S209.	Conference abstract only
5. Marcu IR, Patru S, Bighea AC. Efficacy of physical exercise programcombined with analgesic medication in patients with knee osteoarthritis. *Osteoporosis International.* 2020 2020;31:S393.	Conference abstract only
6. Strand V, Bergman M, Parenti D, Nezzer J, Young C. A phase 3 randomized controlled trial of lower-dose diclofenac capsules in patients with osteoarthritis pain: Impact on patient-reported outcomes. *Osteoarthritis and Cartilage.* 2014 2014;22:S392-S393.	Conference abstract only
7. Van Tunen JAC, Van Der Leeden M, Bos W, et al. Exercise therapy in patients with knee osteoarthritis and severe pain is enabled by optimization of analgesics-a feasibility study. *Annals of the Rheumatic Diseases.* 2015 2015;74:1318.	Conference abstract only
8. Van Tunen JA, Van Der Leeden M, Bos W, et al. Optimization of analgesics allows patients with knee osteoarthritis and severe pain to participate in exercise therapy. *Osteoarthritis and Cartilage.* 2015 2015;23:A370-A371.	Conference abstract only
9. Altman RD, Zinsenheim JR, Temple AR, Schweinle JE. Three-month efficacy and safety of acetaminophen extended-release for osteoarthritis pain of the hip or knee: a randomized, double-blind, placebo-controlled study. *Osteoarthritis and cartilage.* 2007;15(4):454-461	Wrong outcome
10. Casale R, Damiani C, Rosati V, Sarzi-Puttini P, Atzeni F, Nica AS. Efficacy of a comprehensive rehabilitation programme combined with pharmacological treatment in reducing pain in a group of OA	Wrong outcome

patients on a waiting list for total joint replacement. *Clinical and Experimental Rheumatology.* 2012;30(2):233-239.	
11. Olejarova M, Svobodova R, Jarosova H, et al. Efficacy evaluation of nonpharmacological treatment (regular exercise), pharmacotherapy (glucosamine sulphate, GS Condro Forte) and the combination of both methods in symptomatic osteoarthritis of the knee. Results of open, randomized, controlled study. *Ceska Revmatologie.* 2008;16(4):153-160.	Wrong drug
12. Scott LJ, Lamb HM. Rofecoxib. *Drugs.* 1999;58(3):499-497.	Wrong drug
13. Shen H, Sprott H, Aeschlimann A, et al. Analgesic action of acetaminophen in symptomatic osteoarthritis of the knee. *Rheumatology (Oxford, England).* 2006;45(6):765-770.	No placebo group
14. Enteshari-Moghaddam A, Azami A, Habibzadeh A, Isazadehfar K, Mohebbi H, Jahanpanah P. Efficacy of duloxetine and gabapentin in pain reduction in patients with knee osteoarthritis. *Clinical Rheumatology.* 2019;38(10):2873-2880.	No placebo group
15. Kern K-U, Sohns M, Heckes B, Elling C. Tapentadol prolonged release for severe chronic osteoarthritis pain in the elderly: Improvements in daily functioning and quality of life. *Pain Management.* 2020;10(2):85-95.	No placebo group
16. Giansiracusa JE, Donaldson MS, Koonce ML, Lefton TE, Ruoff GE, Brooks CD. Ibuprofen in osteoarthritis. *Southern medical journal.* 1977;70(1):49-52.	Wrong population
17. Markenson JA, Croft J, Zhang PG, Richards P. Treatment of persistent pain associated with osteoarthritis with controlled-release oxycodone tablets in a randomized controlled clinical trial. *The Clinical journal of pain.* 2005;21(6):524-535.	Wrong population
18. Royer GL, Jr., Moxley TE, Hearron MS, Miyara A, Donovan JF. A six-month double-blind trial of ibuprofen and indomethacin in osteoarthritis. *Current therapeutic research, clinical and experimental.* 1975;17(3):234-248.	Wrong population
19. Woodhouse LJ. Do ibuprofen or glucosamine in addition to resistance exercise training improve muscle strength in patients with knee osteoarthritis? *Clinical Journal of Sport Medicine.* 2012;22(3):290-292.	Commentary
20. Ehrich EW, Bolognese JA, Watson DJ, Kong SX. Effect of rofecoxib therapy on measures of health-related quality of life in patients with osteoarthritis. *The American journal of managed care.* 2001;7(6):609-616	Wrong drug
21. Scott DL, Berry H, Capell H, et al. The long-term effects of non-steroidal anti-inflammatory drugs in osteoarthritis of the knee: a randomized placebo-controlled trial. *Rheumatology (Oxford, England).* 2000;39(10):1095-1101.	Rescue medication in the control group
22. Rauck R, Rapoport R, Thipphawong J. Results of a Double-blind, Placebo-controlled, Fixed-dose Assessment of Once-daily OROS Hydromorphone ER in Patients with Moderate to Severe Pain Associated with Chronic Osteoarthritis. *Pain Practice.* 2013;13(1):18-29.	Rescue medication in the control group
23. Thorne C, Beaulieu AD, Callaghan DJ, et al. A randomized, double-blind, crossover comparison of the efficacy and safety of oral controlled-release tramadol and placebo in patients with painful osteoarthritis. *Pain research & management.* 2008;13(2):93-102.	Rescue medication in the control group

24. Vorsanger G, Xiang J, Jordan D, Farrell J. Post hoc analysis of a randomized, double-blind, placebo-controlled efficacy and tolerability study of tramadol extended release for the treatment of osteoarthritis pain in geriatric patients. *Clinical therapeutics.* 2007;29 Suppl:2520-2535.	Secondary analysis of Gana et al. 2006
25. Hair PI, Curran MP, Keam SJ. Tramadol extended-release tablets. *Drugs.* 2006;66(15):2017-2030.	Review
26. Peloso PM, Bellamy N, Bensen W, et al. Double blind randomized placebo control trial of controlled release codeine in the treatment of osteoarthritis of the hip or knee. *The Journal of rheumatology.* 2000;27(3):764-771.	Rescue medication in the control group
27. Bingham CO, 3rd, Sebba AI, Rubin BR, et al. Efficacy and safety of etoricoxib 30 mg and celecoxib 200 mg in the treatment of osteoarthritis in two identically designed, randomized, placebo-controlled, non-inferiority studies. *Rheumatology (Oxford, England).* 2007;46(3):496-507	Rescue medication in the control group
28. Katz N, Sun S, Johnson F, Stauffer J. ALO-01 (morphine sulfate and naltrexone hydrochloride) extended-release capsules in the treatment of chronic pain of osteoarthritis of the hip or knee: pharmacokinetics, efficacy, and safety. *The journal of pain : official journal of the American Pain Society.* 2010;11(4):303-311.	Rescue medication in the control group
29. Leung AT, Malmstrom K, Gallacher AE, et al. Efficacy and tolerability profile of etoricoxib in patients with osteoarthritis: A randomized, double-blind, placebo and active-comparator controlled 12-week efficacy trial. *Current medical research and opinion.* 2002;18(2):49-58.	Rescue medication in the control group
30. Reed K, Collaku A, Moreira S. Efficacy and safety of twice daily sustained-release paracetamol formulation for osteoarthritis pain of the knee or hip: a randomized, double-blind, placebo-controlled, twelve-week study. *Current medical research and opinion.* 2018;34(4):689-699.	Rescue medication in the control group
31. Schnitzer TJ, Beier J, Geusens P, et al. Efficacy and safety of four doses of lumiracoxib versus diclofenac in patients with knee or hip primary osteoarthritis: a phase II, four-week, multicenter, randomized, double-blind, placebo-controlled trial. *Arthritis and rheumatism.* 2004;51(4):549-557.	Rescue medication in the control group
32. Schnitzer TJ, Kivitz A, Frayssinet H, Duquesroix B. Efficacy and safety of naproxcinod in the treatment of patients with osteoarthritis of the knee: a 13-week prospective, randomized, multicenter study. *Osteoarthritis and cartilage.* 2010;18(5):629-639.	Rescue medication in the control group
33. Silverfield JC, Kamin M, Wu S-C, Rosenthal N, Group C-S. Tramadol/acetaminophen combination tablets for the treatment of osteoarthritis flare pain: a multicenter, outpatient, randomized, double-blind, placebo-controlled, parallel-group, add-on study. *Clinical therapeutics.* 2002;24(2):282-297.	Rescue medication in the control group
34. Boyer KA, Angst MS, Asay J, Giori NJ, Andriacchi TP. Sensitivity of gait parameters to the effects of anti-inflammatory and opioid treatments in knee osteoarthritis patients. *Journal of orthopaedic research :*	Rescue medication in the control group

official publication of the Orthopaedic Research Society. 2012;30(7):1118-1124.	
35. Van Tunen JA, Van Der Leeden M, Bos W, et al. Optimization of analgesics allows patients with knee osteoarthritis and severe pain to participate in exercise therapy. *Osteoarthritis and Cartilage*. 2015 2015;23:A370-A371.	Wrong outcome
36. Petersen SG, Saxne T, Heinegard D, et al. Glucosamine but not ibuprofen alters cartilage turnover in osteoarthritis patients in response to physical training. *Osteoarthritis and cartilage*. 2010 2010;18(1):34-40.	Wrong outcome
37. Petersen SG, Beyer N, Hansen M, et al. Nonsteroidal anti-inflammatory drug or glucosamine reduced pain and improved muscle strength with resistance training in a randomized controlled trial of knee osteoarthritis patients. *Archives of physical medicine and rehabilitation.* 2011 2011;92(8):1185-1193.	Wrong outcome
38. Petersen SG, Miller BF, Hansen M, Kjaer M, Holm L. Exercise and NSAIDs: effect on muscle protein synthesis in patients with knee osteoarthritis. *Medicine and science in sports and exercise*. 2011 2011;43(3):425-431.	Wrong outcome
Identified in citation searching from systematic reviews	
1. Pincus T, Koch G, Lei H, et al. Patient Preference for Placebo, Acetaminophen (paracetamol) or Celecoxib Efficacy Studies (PACES): two randomised, double blind, placebo controlled, crossover clinical trials in patients with knee or hip osteoarthritis. *Ann Rheum Dis*. Aug 2004;63(8):931-939.	Wrong outcome
2. Roth SH, Fleischmann RM, Burch FX, et al. Around-the-clock, controlled-release oxycodone therapy for osteoarthritis-related pain: placebo-controlled trial and long-term evaluation. Arch Intern Med. Mar 27 2000;160(6):853-860.	Wrong population
3. Malonne H, Coffiner M, Sonet B, Sereno A, Vanderbist F. Efficacy and tolerability of sustained-release tramadol in the treatment of symptomatic osteoarthritis of the hip or knee: a multicenter, randomized, double-blind, placebo-controlled study. *Clin Ther.* Nov 2004;26(11):1774-1782.	Wrong outcome
4. Caldwell JR, Rapoport RJ, Davis JC, et al. Efficacy and safety of a once-daily morphine formulation in chronic, moderate-to-severe osteoarthritis pain: results from a randomized, placebo-controlled, double-blind trial and an open-label extension trial. *J Pain Symptom Manage.* Apr 2002;23(4):278-291.	Rescue medication in the control group
5. Boswell DJ, Ostergaard K, Philipson RS, et al. Evaluation of GW406381 for treatment of osteoarthritis of the knee: two randomized, controlled studies. *Medscape J Med.* 2008;10(11):259.	Rescue medication in the control group
6. Fleischmann R, Sheldon E, Maldonado-Cocco J, Dutta D, Yu S, Sloan VS. Lumiracoxib is effective in the treatment of osteoarthritis of the knee: a prospective randomized 13-week study versus placebo and celecoxib. Clin Rheumatol. Feb 2005;25(1):42-53.	Rescue medication in the control group
7. Lehmann R, Brzosko M, Kopsa P, et al. Efficacy and tolerability of lumiracoxib 100 mg once daily in knee osteoarthritis: a 13-week, randomized, double-blind study vs. placebo and celecoxib. *Curr Med Res Opin.* Apr 2005;21(4):517-526.	Rescue medication in the control group

8. Baerwald C, Verdecchia P, Duquesroix B, Frayssinet H, Ferreira T. Efficacy, safety, and effects on blood pressure of naproxcinod 750 mg twice daily compared with placebo and naproxen 500 mg twice daily in patients with osteoarthritis of the hip: a randomized, double-blind, parallel-group, multicenter study. *Arthritis Rheum.* Dec 2010;62(12):3635-3644.	Rescue medication in the control group
9. Sawitzke AD, Shi H, Finco MF, et al. Clinical efficacy and safety of glucosamine, chondroitin sulphate, their combination, celecoxib or placebo taken to treat osteoarthritis of the knee: 2-year results from GAIT. *Ann Rheum Dis.* Aug 2010;69(8):1459-1464.	Rescue medication in the control group
10. Schnitzer TJ, Dattani ID, Seriolo B, et al. A 13-week, multicenter, randomized, double-blind study of lumiracoxib in hip osteoarthritis. *Clin Rheumatol.* Nov 2011;30(11):1433-1446.	Rescue medication in the control group
11. Schnitzer TJ, Hochberg MC, Marrero CE, Duquesroix B, Frayssinet H, Beekman M. Efficacy and safety of naproxcinod in patients with osteoarthritis of the knee: a 53-week prospective randomized multicenter study. *Semin Arthritis Rheum.* Feb 2011;40(4):285-297.	Rescue medication in the control group

F

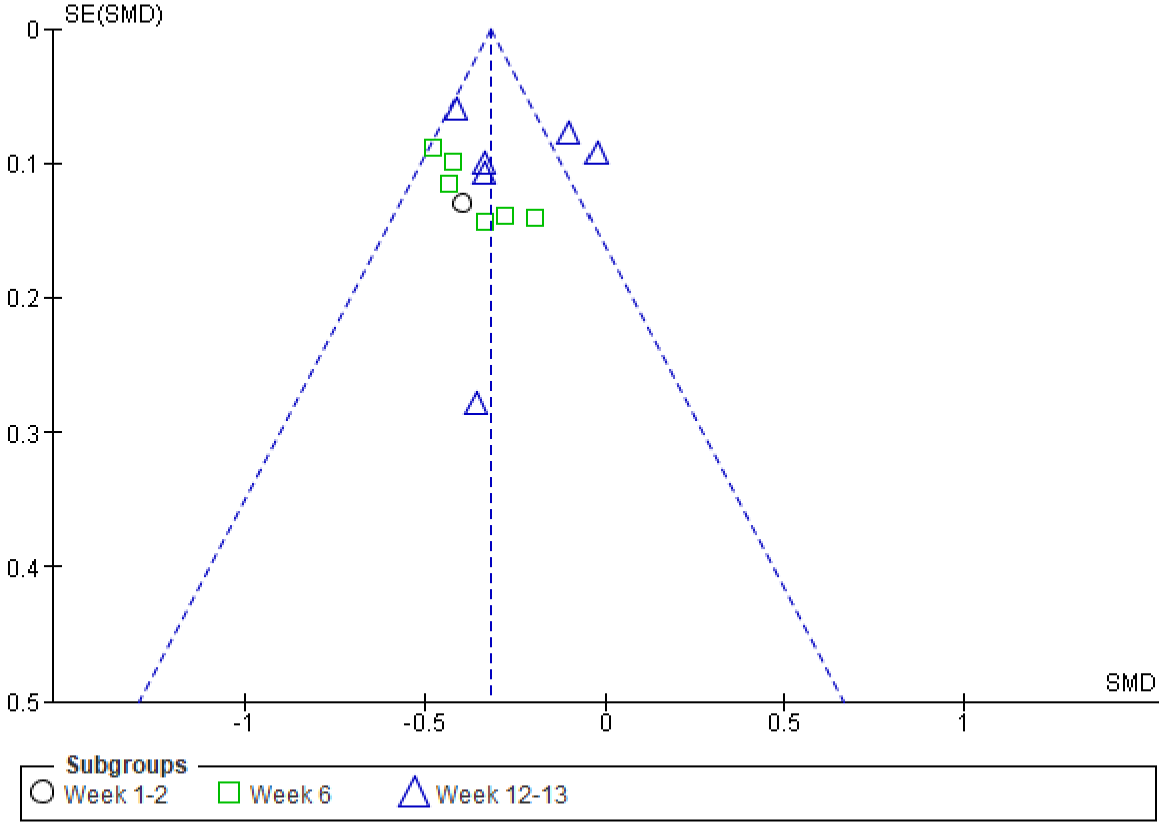

Supplementary file 3

Funnel plot illustrating risk of publication bias for the evidence for effect of non-steroidal drugs on self-reported physical function in people with hip or knee osteoarthritis.
